# Utility of endoscopic ultrasound-guided fine-needle aspiration of regional lymph nodes that are proximal to and far from the primary distal esophageal carcinoma

**DOI:** 10.18632/oncotarget.18119

**Published:** 2017-05-23

**Authors:** Yusuke Shimodaira, Rebecca S. Slack, Kazuto Harada, Manoop S. Bhutani, Elena Elimova, Gregg A. Staerkel, Nour Sneige, Jeremy Erasmus, Hironori Shiozaki, Nikolaos Charalampakis, Venkatram Planjery, Dilsa Mizrak Kaya, Fatemeh G. Amlashi, Mariela A. Blum, Heath D. Skinner, Bruce D. Minsky, Dipen M. Maru, Wayne L. Hofstetter, Stephen G. Swisher, Jeannette E. Mares, Jane E. Rogers, Quan D. Lin, William A. Ross, Brian Weston, Jeffrey H. Lee, Jaffer A. Ajani

**Affiliations:** ^1^ Department of Gastrointestinal Medical Oncology, University of Texas M.D. Anderson Cancer Center, Houston, Texas, USA; ^2^ Department of Biostatistics, University of Texas M.D. Anderson Cancer Center, Houston, Texas, USA; ^3^ Department of Gastroenterology, University of Texas M.D. Anderson Cancer Center, Houston, Texas, USA; ^4^ Department of Anatomic Pathology, University of Texas M.D. Anderson Cancer Center, Houston, Texas, USA; ^5^ Department of Pathology, University of Texas M.D. Anderson Cancer Center, Houston, Texas, USA; ^6^ Department of Diagnostic Radiology, University of Texas M.D. Anderson Cancer Center, Houston, Texas, USA; ^7^ Department of Radiation Oncology, University of Texas M.D. Anderson Cancer Center, Houston, Texas, USA; ^8^ Department of Thoracic and Cardiovascular Surgery, University of Texas M.D. Anderson Cancer Center, Houston, Texas, USA; ^9^ Department of Pharmacy, University of Texas M.D. Anderson Cancer Center, Houston, Texas, USA

**Keywords:** esophageal carcinoma, fine-needle aspiration, endoscopic ultrasound, upper mediastinal lymph nodes, positron emission tomography

## Abstract

Implications of assessing the proximal and far para-tracheal or sub-carinal nodes (para-tracheal [PTN] or sub-carinal [SCN]) associated with lower primary esophageal carcinomas (ECs) are unclear. To evaluate the value of endoscopic ultrasound guided fine-needle aspiration (EUS-FNA) for PTN and SCN, we analyzed results by positron emission tomography (PET) avidity, 4 EUS node malignancy features, and EUS-FNA results in all patients with Siewert’s I or II EC. Of 133 patients (PTN, *n*=102; SCN, *n*=31) with EUS-FNA, 47 (35%) patients had malignant node, leading to treatment modifications. EUS-FNA diagnosed significantly more patients with malignant nodes (*p*=0.02) even when PET and EUS features were combined. Among 94 PET-negative and EUS-negative patients, 9 (10%) had malignant EUS-FNA. At a minimum follow-up of 1 year, only 3 (5%) of 62 patients with benign EUS-FNA had evidence of malignancy in the nodal area of prior EUS-FNA. Patients with malignant EUS-FNA independently had a much shorter overall survival (OS) than those with benign EUS-FNA (*p*<0.001). Our data suggest that a benign EUS-FNA is highly accurate and need not be pursued further. However, malignant EUS-FNA of PTN/SCN was independently prognostic, conferred a shorter OS, and altered the management of 35% of patients.

## INTRODUCTION

**Figure 1:**
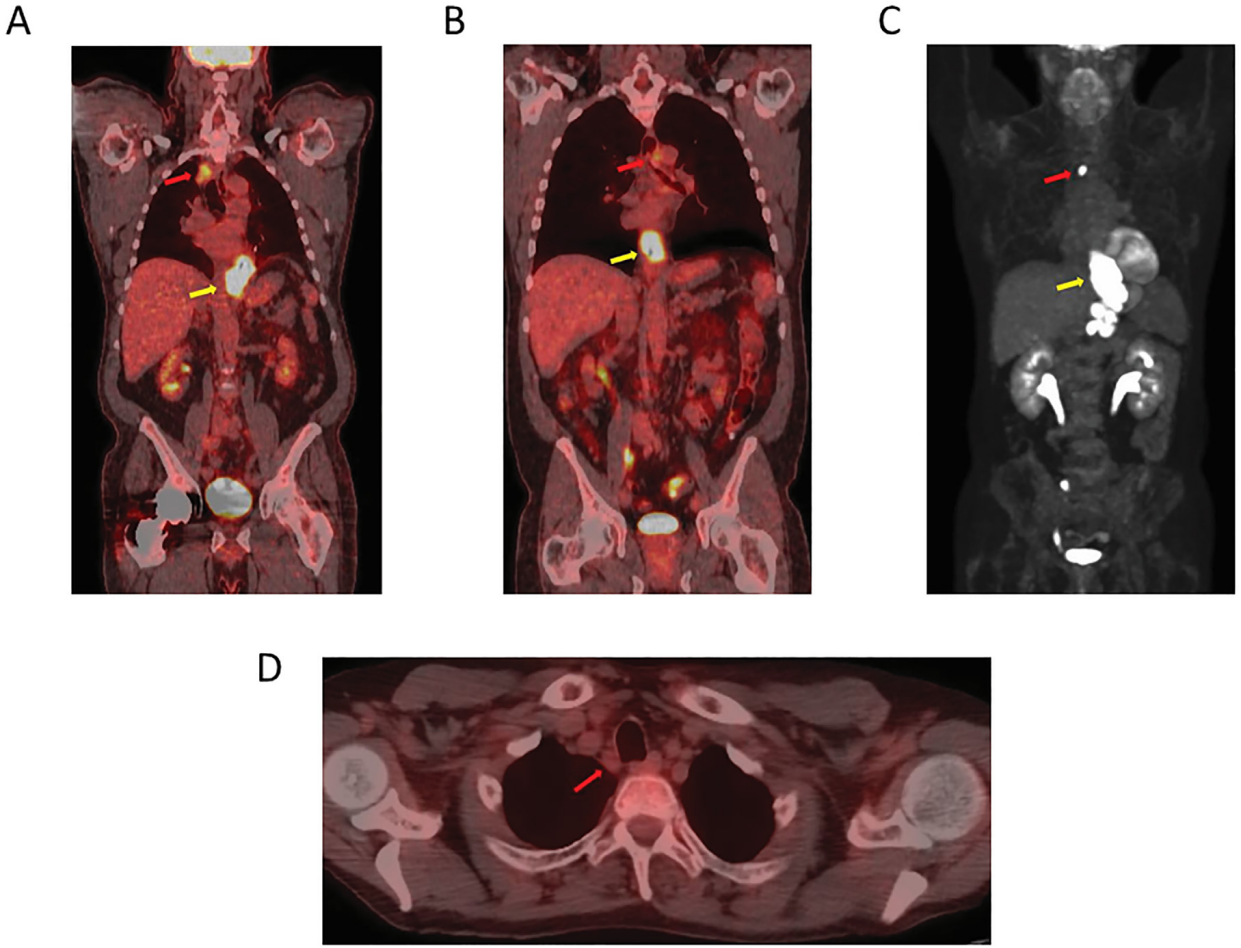
A PET-CT image showing a lower esophageal/GEJ primary cancer **A.** An avid high right upper mediastinal node. **B.** An avid high left upper mediastinal node. **C.** An avid high mediastinal node (towards right side). **D.** A right paratracheal non-avid node.

Despite research advances, esophageal carcinoma (EC) remains a significant health burden around the world with an estimated 455,800 new cases and 400,200 deaths occurring in 2012 [1]. Clinical staging of EC is central in making the choice of initial therapy that ranges from endoscopic resection to multimodality therapy [2-5]. The presence of malignant nodes in EC imparts a very poor prognosis [6]. Although, endoscopic ultrasound (EUS), EUS-guided fine-needle aspiration (EUS-FNA), and other elements of good baseline imaging (including positron emission tomography [PET]) may be useful [7], some debate continues, particularly regarding the need for EUS itself when EC is obviously large on the imaging studies or if a patient has stricture/dysphagia [8-11], EUS is a reasonable tool for establishing the T stage of EC and a little less reliable for N stage compared to its performance with T staging [12]. Prior to developing an initial therapy plan such as chemoradiation followed by surgery (trimodality; TMT), it may not be necessary to incorporate EUS/EUS-FNA in initial staging in the vicinity of the primary (some argue that the treatment choice remains the same with or without EUS). However, the value of EUS-FNA is unclear when nodes are proximal to and far (para-tracheal [PTN] or subcarinal [SCN]) from a lower esophageal primary (Figures 1A-1D). Computed tomography (CT) and PET have lower sensitivity and specificity than EUS for T stage and N stage; therefore PET especially finds its use in the detection of metastatic disease [13-18]. EUS has its own set of limitations [14-16, 19, 20]. The skill level of the endoscopist and experience (volume) cannot be overemphasized. Not many retrospective reports and only 1 prospective trial have addressed the value of EUS-FNA focused on EC, in addition, none focused on the proximal nodes as we describe in this report [5, 21-23]. Our focus is quite different in that we were not necessarily emphasizing to characterize the nodes in the vicinity of the primary (including the celiac region) but we focused on PTN/SCN. Since a PET (or CT) is reviewed prior to EUS at our institution, it affords an added opportunity to carefully review the thoracic inlet and areas far above the lower EC. If such nodes are identified by imaging or found by EUS, our policy has been to attempt EUS-FNA. Detection of malignant PTN/SCN could significantly alter the management. Whether the outcome of patients differs based on the treatment modifications guided by EUS-FNA results is not known. In addition, reliability of a benign result of EUS-FNA in this setting is unknown. The purpose of this study was to investigate the effectiveness of EUS-FNA in a large cohort of patients by addressing the following issues: (1) How often EUS-FNA findings change treatment choices? (2) Whether EUS-FNA is a reliable diagnostic tool for these nodes? (3) Do the outcomes change based on the EUS-FNA results, and (4) Is EUS-FNA safe? To our knowledge, similar data have not been reported in the literature.

## RESULTS

### Patient characteristics

Patient characteristics are listed in Table 1. Patients were predominantly Caucasians (86%), men (89%), with adenocarcinoma (95%), and Eastern Cooperative Oncology Group (ECOG) performance status of 0 or 1 (94%).

**Table 1:** Patient characteristics and EUS features

		All	EUS-FNA-	EUS-FNA+	
Variable	Patient Characteristics	*N* (%)	*N* (%)	*N* (%)	*P*-value
All		133 (100%)	86 (65%)	47 (35%)	
Age at Dx median(min,max)	*N* = 133	64.0 (32.0, 84.0)	64.5 (32.0, 84.0)	64.0 (44.0, 84.0)	0.98
Gender					0.58
	F	14 (11%)	10 (71%)	4 (29%)	
	M	119 (89%)	76 (64%)	43 (36%)	
Race					0.63
	White	114 (86%)	74 (65%)	40 (35%)	
	Hispanic	6 (5%)	5 (83%)	1 (17%)	
	Other	13 (10%)	7 (54%)	6 (46%)	
ECOG					0.79
	0	43 (32%)	30 (70%)	13 (30%)	
	1	82 (62%)	51 (62%)	31 (38%)	
	2	7 (5%)	4 (57%)	3 (43%)	
	3	1 (1%)	1 (100%)	0 (0%)	
Histology					>0.99
	Adenocarcinoma	126 (95%)	81 (64%)	45 (36%)	
	Squamous cell ca.	7 (5%)	5 (71%)	2 (29%)	
Grade					0.55
	Well-differentiated	2 (2%)	2 (100%)	0 (0%)	
	Moderately differentiated	64 (48%)	40 (63%)	24 (38%)	
	Poorly differentiated	67 (50%)	44 (66%)	23 (34%)	
Baseline Stage*					NT
	I	11 (8%)	11 (100%)	0 (0%)	
	II	34 (26%)	31 (91%)	3 (9%)	
	III	79 (59%)	41 (52%)	38 (48%)	
	IV*	7 (5%)	2 (29%)	5 (71%)	
LN*					NT
	N-	43 (32%)	43 (100%)	0 (0%)	
	N+	89 (67%)	43 (48%)	46 (52%)	
Tumor Length median(min,max)	*N* = 133	5.0 (0.5, 15.0)	4.5 (0.5, 13.0)	6.0 (1.0, 15.0)	**0.001**
EUS Features					**<0.001**
	No features	8 (6%)	7 (88%)	1 (13%)	
	1	42 (32%)	38 (90%)	4 (10%)	
	2	45 (34%)	35 (78%)	10 (22%)	
	3	11 (8%)	6 (55%)	5 (45%)	
	Positive (4)	27 (20%)	0 (0%)	27 (100%)	
Treatment					NT
	Surgery only	3 (2%)	3 (100%)	0 (0%)	
	CRT + Surgery	53 (40%)	40 (75%)	13 (25%)	
	Definitive CRT	47 (35%)	27 (57%)	20 (43%)	
	Chemotherpy	14 (11%)	4 (29%)	10 (71%)	
	EMR	10 (8%)	10 (100%)	0 (0%)	
	Unknown	6 (4%)	2 (33%)	4 (66%)	

NT=Not tested. Since EUS-FNA+ results by definition cannot be associated with Stage I or LN N0, no test was performed to identify differences in status or location of lymph nodes.

*2 patients are missing baseline stage, 1 EUS-FNA- and 1 EUS-FNA+. 1 EUS-FNA+ patient is missing LN status. *Localized stage 4 by AJCC 6

Longer tumor length was associated with malignant EUS-FNA (the median tumor length was 4.5 cm for benign FNA and 6 cm for malignant FNA; *p* = 0.001).

### EUS-FNA results in context of EUS on PTN or SCN

Table 1 shows that all 27 EUS positive patients also had malignant EUS-FNA (100%). EUS was negative (with none to three of the four malignant echo features) in 106 patients. Among these patients, EUS-FNA was malignant in only 20 (19%). Nodes with a higher number of echo features had higher rates of being malignant by EUS-FNA (13%, 10%, 22%, 45%, and 100% malignant nodes with 0,1, 2, 3, and 4 malignant echo features, respectively; *p* < 0.001).

### EUS-FNA results in the context of PET avidity of PTN or SCN

Among 133 patients, PET and EUS-FNA were both positive in 29 and both negative in 85 patients. PET identified as being positive in 1 patient but had a benign EUS-FNA result (patient followed already for 16 months without recurrence), while 18 patients had malignant EUS-FNA results when was PET negative. EUS-FNA identified significantly more patients with malignant nodes than did positive PET (*p* < 0.001; Table 2).

**Table 2:** Crosstabulation of PET vs. EUS-FNA results of PTN or SCN

		EUS-FNA		p-value
		Positive	Negative	<0.001
PET	Positive	29	**1**	
	Negative	**18**	85	

### EUS-FNA results when PET and EUS findings are combined

Positive PET and/or positive EUS were noted in 39 patients and EUS-FNA diagnosed malignant nodes in 38 (97%; *p* = 0.02) patients, however, negative PET and negative EUS ( < 4 echo features) were noted in 94 patients. Nevertheless, EUS-FNAs were performed in all 94 patients and 9 (10%) had malignant nodes by EUS-FNA (*p* = 0.02; Table 3).

**Table 3:** Crosstabulation of PET+EUS vs. EUS-FNA results of PTN or SCN

		EUS-FNA		*p*-value
		Positive	Negative	0.02
PET+EUS*	Positive	38	1	
	Negative	9	85	

*The LN with SUV avidity above physiologic background was count as PET positive otherwise negative. The LN with all of these four characteristics by EUS was taken as EUS positive; (1) size 10 mm or larger; (2) round shape; (3) homogeneous hypoechoic pattern; (4) sharp or distinct borders. The LN diagnosed as positive by both PET and EUS or, by at least one of the two exams is counted as PET+EUS positive, while the LN detected as no malignancy by both PET and EUS is counted as PET+EUS negative.

### Impact of the EUS-FNA results on treatment modifications

In 47 of 133 patients with positive EUS-FNA, only 30 (22.6%) patients had change in their radiation field. See the comment above describing the changes made in the radiation field.

### Outcome of the patients with malignant nodes by EUS-FNA

Among 47 patients with malignant nodes by EUS-FNA, 13 patients were treated with TMT, 21 patients were treated with definitive chemoradiation, 9 patients were treated with chemotherapy only, and 4 patients receive no therapy. PTN/SCN was included if patients received radiation therapy. Post-therapy relapse-free survival (RFS) could be calculated in 37 patients but could not be calculated in 10 patients because: one patient was lost to follow-up, 5 patients were found to have distant metastases, and 4 patients did not receive any therapy. The median follow-up time was 23.3 months (95% confidence interval [CI]: 18.9 to 34.4 months) with the longest follow-up at 39.9 months. 9 (24%) of 37 patients were without recurrence at last follow-up, 2 (4%) were diagnosed with local recurrence, 3 (8%) were diagnosed with both local and distant metastases, 20 (54%) had distant metastases, and 3 (8%) died without a known cause. The median RFS time was only 5.3 months (95% CI: 2.3 to 8.4).

### Minimum of 1-year follow-up of patients with benign nodes by EUS-FNA

62 patients, who had benign nodes by EUS-FNA (once benign, PTN/SCN were not included in the radiation field), were followed for more than 1 year from the date of EUS-FNA. Among these, 33 patients were treated by TMT, 17 patients received definitive chemoradiation, 11 patients underwent endoscopic mucosal resection, and 1 patient received chemotherapy only. Only 3 patients treated with chemoradiation followed by surgery developed histologically confirmed malignancy in the area of prior EUS-FNA. Thus only 3 of 62 patients (5%; 95% CI [1%, 13%]) had malignant LN(s) in the area(s) of prior EUS-FNA and all others ha no evidence of malignancy in that area.

### OS/RFS and multivariate analysis

**Figure 2:**
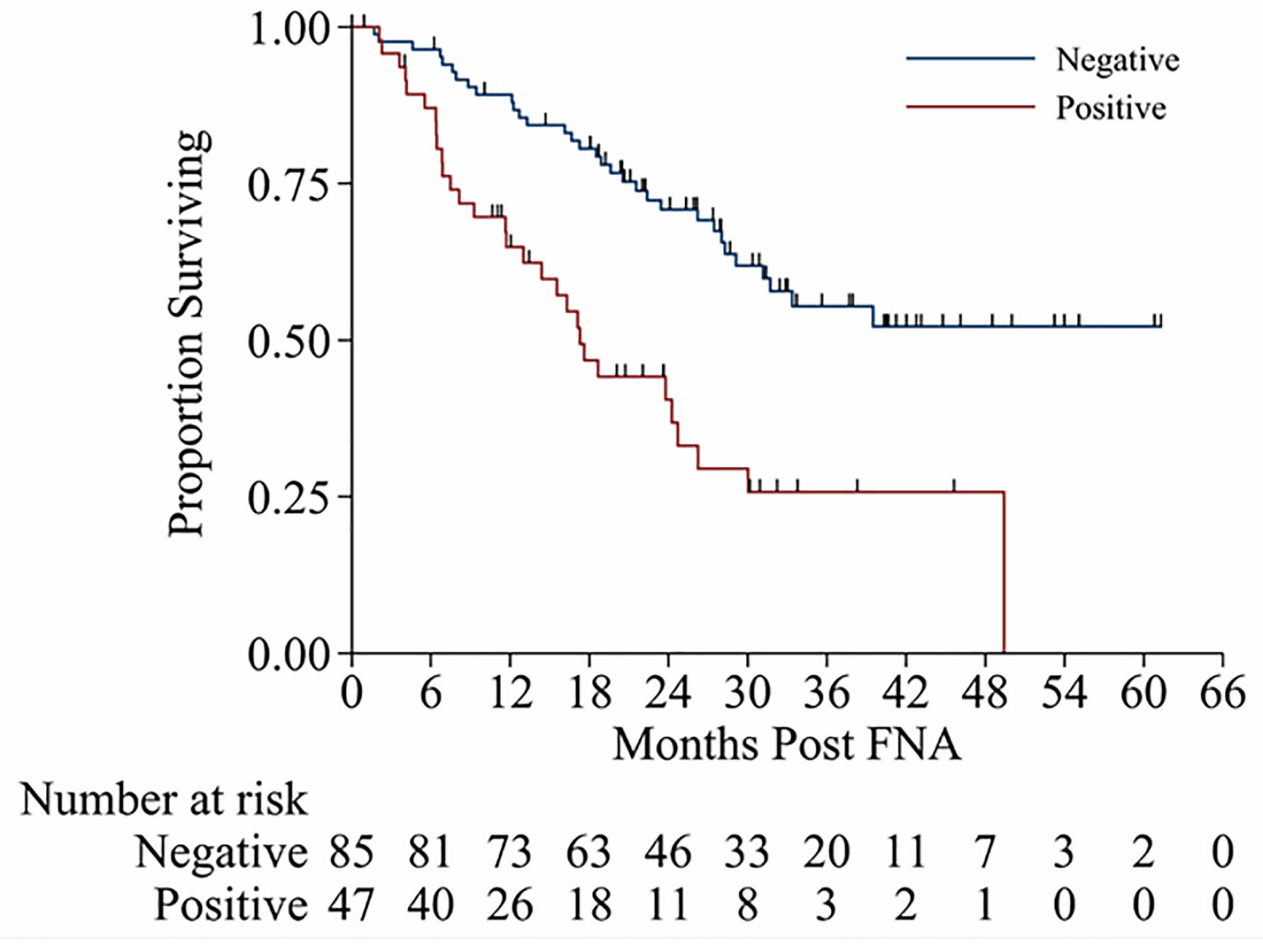
Kaplan-Meier survival curves from time of EUS-FNA for patients with malignant EUS-FAN and benign EUS-FNA

Patients with malignant PTN/SCN by EUS-FNA had a shorter overall survival (OS) than those patients with benign PTN/SCN by EUS-FNA (2-year OS rate of 71% *vs* 41%; *p* = < 0.001; Figure 2). In the multivariate analysis, malignant PTN/SCN was associated with higher hazard ratio (HR) for death (2.4 with 95% CI: 1.3 to 4.4; *p* = 0.005) in the reduced model. Similarly, histologic grade (HR 1.9, 95% CI from 1.1 to 3.3; *p* = 0.02) and clinical LNs (HR 2.7, 95% CI from 1.2 to 5.7; *p* = 0.01) were independent prognosticator of OS, in the reduced model (Table 4).

**Table 4:** Multivariate survival with selected characteristics (N=131 with 60 events)

		Full Model		Reduced Model	
Patient Characteristics		HR (95% CI)	*P*-Value	HR (95% CI)	*P*-Value
Age	Unit=1 year	1.05 (1.02, 1.09)	**0.002**	1.05 (1.02, 1.09)	**0.001**
Gender	Female vs. Male	0.7 (0.2, 2.0)	0.49		
Baseline Stage	III/IV vs. I/II	0.9 (0.2, 3.4)	0.86		
Grade	Poor vs. Well/Mod	2.0 (1.1, 3.4)	**0.02**	1.9 (1.1, 3.3)	**0.02**
LN	N1 vs.N0	3.2 (0.7, 14.3)	0.13	2.7 (1.2, 5.7)	**0.01**
EUS-FNA	Positive vs. Negative	2.2 (1.2, 4.2)	**0.01**	2.4 (1.3, 4.4)	**0.005**

### Patterns of spread in patients who had malignant nodes by EUS-FNA

**Figure 3:**
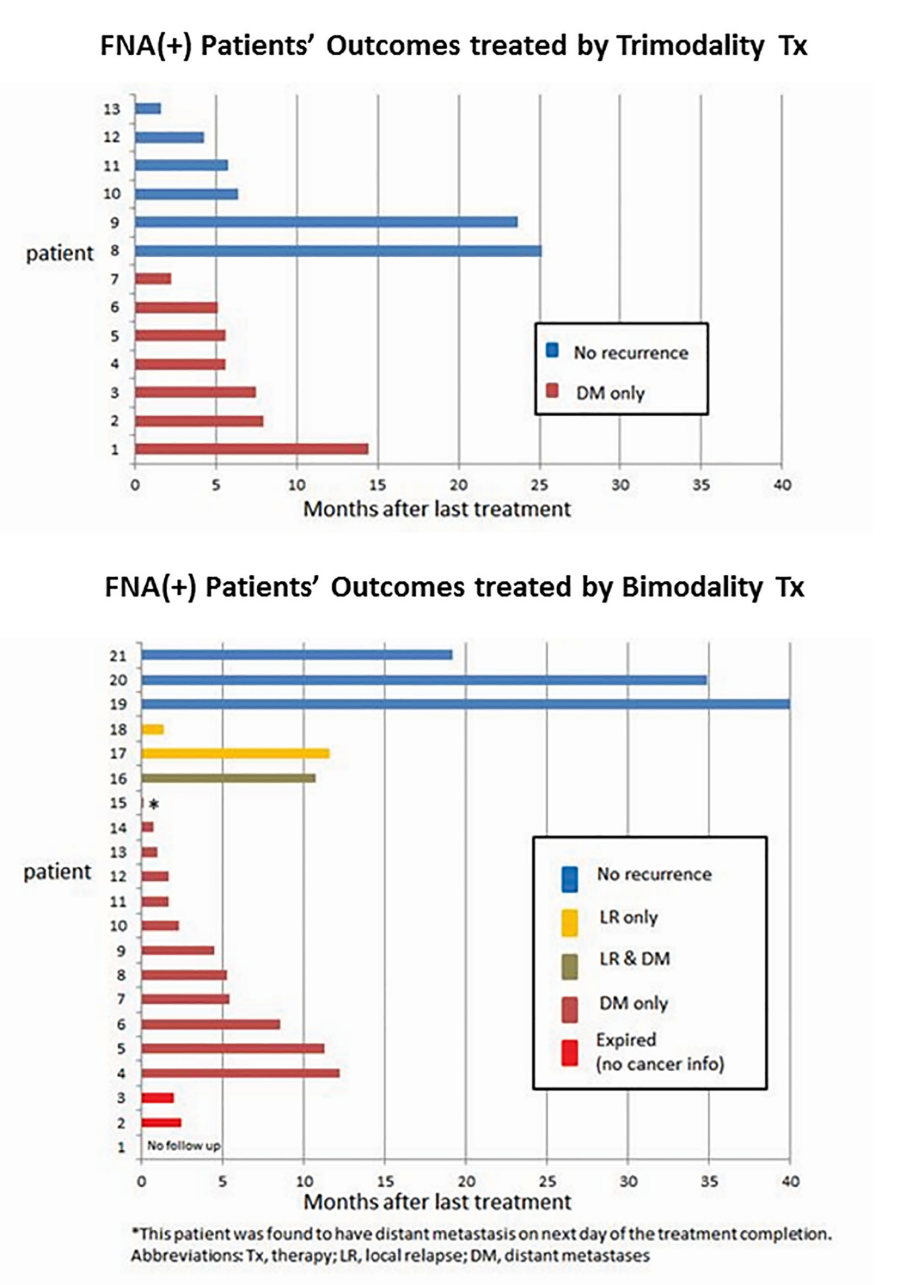
Recurrence patterns for patients treated by trimodality treatment and treated by bimodality treatment

Figures 3 demonstrate the type of relapses experienced by patients who received either TMT or bimodality therapy (Figure 3). Distant metastases were common and TMT patients did not experience local or local-regional relapse.

### Safety

No complications were experienced during the EUS and EUS-FNA procedures described in this report.

## DISCUSSION

This study represents, to our knowledge, the first to assess the value of PTN/SCN EUS-FNA patients with Siewert’s type I or II ECs. Many reports have mentioned the utility of EUS-FNA for LNs of patients with EC [5, 21-23], none have evaluated PTN/SCN. Our results documented several unique findings: (1) review of imaging studies prior to EUS was helpful in identifying proximal nodes that could then be targeted by EUS-FNA, (2) a benign results by EUS-FNA were highly reliable and need not be pursued with additional biopsies and even PET-positive but benign EUS-FNA LNs may not need to be included in the radiation field, (3) higher the number of malignant echo features of LNs, higher was the chance of diagnosing malignant nodes by EUS-FNA (with 100% rate of malignancy by EUS-FNA, if all 4 echo features were present), (4) EUS-FNA identified significantly more patients with malignant nodes than did PET (*p* < 0.001) and this was still significant when PET result and EUS result were combined (*P* = 0.02), (5) patients with malignant PTN/SCN by EUS-FNA had a shorter OS compared to patients with benign PTN/SCN by EUS-FNA (*p* = 0.001) and it was an independent prognosticator of OS, and (6) the longer the length of the tumor, the higher was the chance of malignant nodes by EUS-FNA (*p* = 0.001). Our report only establishes that change in therapy may be needed in patients with malignant proximal nodes by EUS-FNA but does not establish that the altered therapeutic plan is beneficial to such patients.

EUS is highly effective for T staging, if FNA is added, it is also effective for N staging [24]. FNA plays a larger role as it can confirm malignancy or lack of malignance. Current study showed that FNA was positive in 10% of LNs that were negative by PET and EUS. This suggests that an experienced gastroenterologist can decide to perform FNA in lowly suspicious cases. In current study, 57 patients with LNs with negative FNA remained negative after esophagectomy, however some malignant LNs in the resected specimens were not FNAed. Further improvement in clinical staging could identify these nodes in the future. Taken together, flexible treatment strategy according to EUS-FNA result is recommended.

We acknowledge many shortcomings in our results: (1) the retrospective nature of the study, (2) results are from a single large volume institution, therefore, not generalizable, (3) EUS-FNA is not a recommended procedure by many guidelines [7, 25-27]. However, the strengths of our results are: (1) this is the first demonstration of some value of assessing nodes that are proximal to and far (yet regional) from the lower primary EC and (2) we provide evidence that a negative EUS-FNA of PTN/SCN is highly reliable. Our results are compelling and point towards routine use of EUS-FNA in all patients with lower esophageal carcinoma. Additionally, various guidelines should deliberate on this strategy to consider embracing it.

In conclusion, EUS-FNA of proximal nodes in patients with lower EC suggests its feasibility, safety, and accuracy. Patient with lower EC who have malignant proximal nodes have a poor prognosis compared to those who do not. The EUS-FNAs were not associated with any complications.

## PATIENTS AND METHODS

### Patient selection

Patients with EC were identified from our prospectively maintained database in the Department of GI Medical Oncology at The University of Texas MD Anderson Cancer Center between July 2010 and June 2014. From the database for patients with upper gastrointestinal endoscopy, we selected patients who had PTN or SCN noted either by PET or by EUS and underwent FNA. These patients had either Siewert’s type I or II type of EC. Patients with Siewert type III cancer and those with distant metastases were excluded. All patients had a baseline PET (prior to EUS). PTNs are nodal stations 2, 4, and 5, and SCN is LN of station 7 [28] (Station 1 nodes are inaccessible by EGD). PET avidity of PTN/SCN above physiologic level was considered PET-positive. Upon completion of clinical staging, all patients were presented to our multidisciplinary esophageal cancer conference to establish a consensus for initial therapy. Patients were systematically followed as previously reported [29, 30]. EC staging was based on the American Joint Committee on Cancer (AJCC) staging manual, sixth edition [28]. The institutional review board approved this analysis.

### EUS-FNA

All EUS-FNA procedures were performed by 4 experienced endosonographers. EUS staging was established using a radial scanning endoscope (Olympus GF-UM 130, GF-UM 160; Olympus America Inc.) and/or a linear array endoscope (Olympus/Aloka GF-UC-130, GF-UC-160P; Aloka Medical Device, Tokyo, Japan) with a frequency range of 5.0 to 20 megahertz. Previously described LN criteria for malignant involvement were adopted for the classification of LNs [31, 32]. These criteria were applied, and recorded for all LNs imaged and were as follows: (1) size 10 mm or greater; (2) round shape; (3) homogeneous hypoechoic pattern; and (4) sharp or distinct borders. All 4 EUS features must have been observed to designate PTN/SCN LNs as malignant (EUS-positive).

EUS-FNA was performed using a 22-gauge or 25-gauge needle. The instrument was placed in the esophagus lumen opposite the identified LN and the needle was advanced through the normal esophagus wall guided into the target site using real-time ultrasound. One to three passes were taken with or without suction at the discretion of endoscopist. An on-site cytologist ensured the adequacy of the specimen before terminating the procedure. Malignant cells seen in the EUS-FNA were designated as EUS-FNA-malignant. When no malignant cells were seen, confirming lymphoid tissue in the EUS-FNA, prior to concluding the case, ensured adequacy of lymph node sampling.

### Treatment strategy

Basically we treated according to the National Comprehensive Cancer Network (NCCN) Guidelines [7]. Patients with Stage I EC underwent surgery without any preoperative treatment. Patients with resectable advanced EC had chemoradiotherapy consisting of radiation and concurrent chemotherapy (with or without induction chemotherapy). Approximately 5 to 6 weeks after the completion of chemoradiotherapy, preoperative restaging were performed. All patients were encouraged to undergo surgery after chemoradiotherapy, but some patients with clinical CR who declined surgery were surveyed. Proximal margin of the radiation fields included LN station 7 regardless of FNA results. In cases where the FNA was positive the following lymph stations received radiation: 2, 4, and 5. This change is strategy was solely based on positive FNA.

### Statistical analysis

The association of patient and tumor characteristics with EUS-FNA outcome was tested with *t*-tests for continuous variables (age and tumor length) and by exact chi-square tests for categorical variables due to small sample sizes. Patients were identified as being positive for disease by PET+EUS if either PET or EUS is positive. A 2x2 table of PET+EUS *vs*. EUS-FNA was created and McNemar’s test, with exact calculations was used to test whether EUS-FNA was different than PET+EUS using a 2-sided 5% significance level. Among patients with negative EUS-FNA findings, the true negative and positive statuses were examined using surgical findings and further follow-up scans. Any positive findings during less than 12 months from EUS-FNA would count as a false negative at the time of EUS-FNA. To be true negative, the patient had to be negative and have follow-up for at least 12 months. Patients who remained negative but had information less than 12 months were excluded from this portion of the analyses. The OS was defined as the time from EUS-FNA to death or last follow up (censored). OS was estimated by the methods of Kaplan and Meier and compared between groups. A multivariate analysis including up to 1 variable for every 10 events was implemented using known clinically relevant characteristics (full model). A backwards selection procedure was then carried out requiring EUS-FNA, grade, and LN status to remain in the model. Recurrence-free survival was calculated among patients who were EUS-FNA positive starting at the completion of therapy until documented recurrence/progression, death, or last follow-up (censored). Kaplan-Meier curves were prepared in Stata 13.1 [StatCorp LP, College Station, TX], times to recurrence among EUS-FNA positive patients were plotted in Excel software 14.6.2, and all other analyses were performed in SAS 9.3 [The SAS Institute, Cary, NC].

## Abbreviations

EC: esophageal carcinoma
EUS: endoscopic ultrasound
EUS-FNA: EUS-guided fine-needle aspiration
PET: positron emission tomography
PTN: para-tracheal lymph node
SCN: subcarinal lymph node
CT: computed tomography
TMT: trimodality
RFS: relapse-free survival
HR: hazard ratio
CI: confidence interval
OS: overall survival
